# Altered Fecal Microbiome and Correlations of the Metabolome with Plasma Metabolites in Dairy Cows with Left Displaced Abomasum

**DOI:** 10.1128/spectrum.01972-22

**Published:** 2022-10-12

**Authors:** Zhengzhong Luo, Kang Yong, Qiao Luo, Zhenlong Du, Li Ma, Yixin Huang, Tao Zhou, Xueping Yao, Liuhong Shen, Shumin Yu, Junliang Deng, Zhihua Ren, Yong Zhang, Zuoting Yan, Zhicai Zuo, Suizhong Cao

**Affiliations:** a Department of Clinical Veterinary Medicine, College of Veterinary Medicine, Sichuan Agricultural University, Chengdu, China; b Department of Animal Husbandry & Veterinary Medicine, College of Animal Science and Technology, Chongqing Three Gorges Vocational College, Chongqing, China; c Department of Clinical Veterinary Medicine, College of Veterinary Medicine, Gansu Agricultural University, Lanzhou, China; d Institute of Biodiversity, Animal Health & Comparative Medicine, College of Medical, Veterinary & Life Sciences, University of Glasgow, Glasgow, United Kingdom; e Lanzhou Institute of Animal Husbandry and Veterinary Pharmaceutical, Chinese Academy of Agricultural Sciences, Lanzhou, China; State Key Laboratory of Microbial Resources, Institute of Microbiology, Chinese Academy of Sciences

**Keywords:** left displaced abomasum, dairy cows, gut dysbiosis, host-microbial metabolism, amino acids, cattle, gut microbiota, metabolism

## Abstract

Left displaced abomasum (LDA) in postpartum dairy cows contributes to significant economic losses. Dairy cows with LDA undergo excessive lipid mobilization and insulin resistance. Although gut dysbiosis is implicated, little is known about the role of the gut microbiota in the abnormal metabolic processes of LDA. To investigate the functional links among microbiota, metabolites, and disease phenotypes in LDA, we performed 16S rDNA gene amplicon sequencing and liquid chromatography-tandem mass spectrometry (LC-MS/MS) of fecal samples from cows with LDA (*n* = 10) and healthy cows (*n* = 10). Plasma marker profiling was synchronously analyzed. In the LDA event, gut microbiota composition and fecal metabolome were shifted in circulation with an amino acid pool deficit in dairy cows. Compared with the healthy cows, salicylic acid derived from microbiota catabolism was decreased in the LDA cows, which negatively correlated with *Akkermansia*, *Prevotella*, non-esterified fatty acid (NEFA), and β-hydroxybutyric acid (BHBA) levels. Conversely, fecal taurolithocholic acid levels were increased in cows with LDA. Based on integrated analysis with the plasma metabolome, eight genera and eight metabolites were associated with LDA. Of note, the increases in *Akkermansia* and *Oscillospira* abundances were negatively correlated with the decreases in 4-pyridoxic acid and cytidine levels, and positively correlated with the increases in NEFA and BHBA levels in amino acid deficit, indicating pyridoxal metabolism-associated gut dysbiosis and lipolysis. Changes in branched-chain amino acids implicated novel host-microbial metabolic pathways involving lipolysis and insulin resistance in cows with LDA. Overall, these results suggest an interplay between host and gut microbes contributing to LDA pathogenesis.

**IMPORTANCE** LDA is a major contributor to economic losses in the dairy industry worldwide; however, the mechanisms associated with the metabolic changes in LDA remain unclear. Most previous studies have focused on the rumen microbiota in terms of understanding the contributors to the productivity and health of dairy cows; this study further sheds light on the relevance of the lower gut microbiota and its associated metabolites in mediating the development of LDA. This study is the first to characterize the correlation between gut microbes and metabolic phenotypes in dairy cows with LDA by leveraging multi-omics data, highlighting that the gut microbe may be involved in the regulation of lipolysis and insulin resistance by modulating the amino acid composition. Moreover, this study provides new markers for further research to understand the pathogenesis of the disease as well as to develop effective treatment and prevention strategies.

## INTRODUCTION

The rising demand for global dairy products has prompted an increase in dairy farming (1). However, disease factors, particularly metabolic disease, can affect dairy cow milk production and quality, which restrains the dairy industry (2). Approximately half of all dairy cows experience health challenges during early lactation, including ketosis, hypocalcemia, and a displaced abomasum (2, 3). The latter is a significant risk factor for the high prevalence of postpartum culling in dairy herds (4). Left displaced abomasum (LDA) is more common than right displaced abomasum (5) and contributes to the highest economic loss among all metabolic diseases, accounting for total costs of $432.48 per primiparous cow or $639.51 per multiparous cow (6). LDA is commonly characterized by high circulating non-esterified fatty acids (NEFA) and β-hydroxybutyric acid (BHBA) concentrations, accompanied by low insulin sensitivity (7–9). Accumulating evidence also points to an association between LDA and concurrent ketosis. For example, dairy cows with a circulating BHBA concentration above 1.4 mmol/L had a 2.83- to 6.70-times greater risk of developing LDA in the first week after calving (10, 11). However, the mechanism by which high postpartum NEFA and BHBA levels cause LDA remains unclear.

Traditionally, hypomotility of the abomasum has been considered an important factor in the pathogenesis of LDA (12). The inhibition of abomasum motility is related to a markedly elevated negative energy balance and insulin resistance (9, 12, 13). Cows experiencing excessive negative energy balance are predisposed to developing metabolic diseases because abundant NEFA is produced by lipolysis of the adipose tissue (14). Metabolomics is now widely used to explain the metabolic processes of dairy cows in disease events (15). Metabolic profiles of cows with LDA have demonstrated marked decreases in amino acid levels in the serum or plasma, including tryptophan, leucine, tyrosine, and isoleucine (7, 16), which may be related to a nutrient deficit during the LDA event which reduces the size of the amino acid pool. Thus, knowledge of the amino acid pool and composition can provide insight into the changes in amino acid metabolism in dairy cows with LDA.

Emerging evidence has implicated alterations in the gut microbiota composition and its metabolites in the pathogenesis of several metabolic disorders (17). Dysbiosis of the gut microbiota fuels metabolic inflammatory conditions such as type 2 diabetes, which is characterized by an increased abundance of Escherichia coli and *Bacteroides* spp. in the intestine (17). Moreover, microbial catabolites, such as bile acids, branched-chain amino acids (BCAA), and indole derivatives, participate in the regulation of glucolipid metabolism, insulin resistance, and systemic inflammation (18). Song et al. (19) suggested that the gut microbial composition of cows with LDA was shifted compared with that of healthy cows. However, knowledge regarding the correlation between gut microbiota and metabolism in cows with LDA is lacking.

Therefore, to address this gap in knowledge, we performed 16S rDNA gene amplicon sequencing and untargeted liquid chromatography–tandem mass spectrometry (LC-MS/MS) metabolomic profiling of fecal samples from cows with LDA and healthy dairy cows. The targeted amino acids in the plasma were concurrently analyzed using targeted LC-MS/MS. The fecal microbiome, fecal metabolome, and plasma metabolome data were then integrated to characterize the alterations in metabolism in LDA, as well as to assess potential functional links between gut microbiota, metabolites, and host phenotypes. Ultimately, these data should help provide a better understanding of LDA pathogenesis in dairy cows.

## RESULTS

### Altered fecal microbiome in cows with LDA.

**FIG 1:**
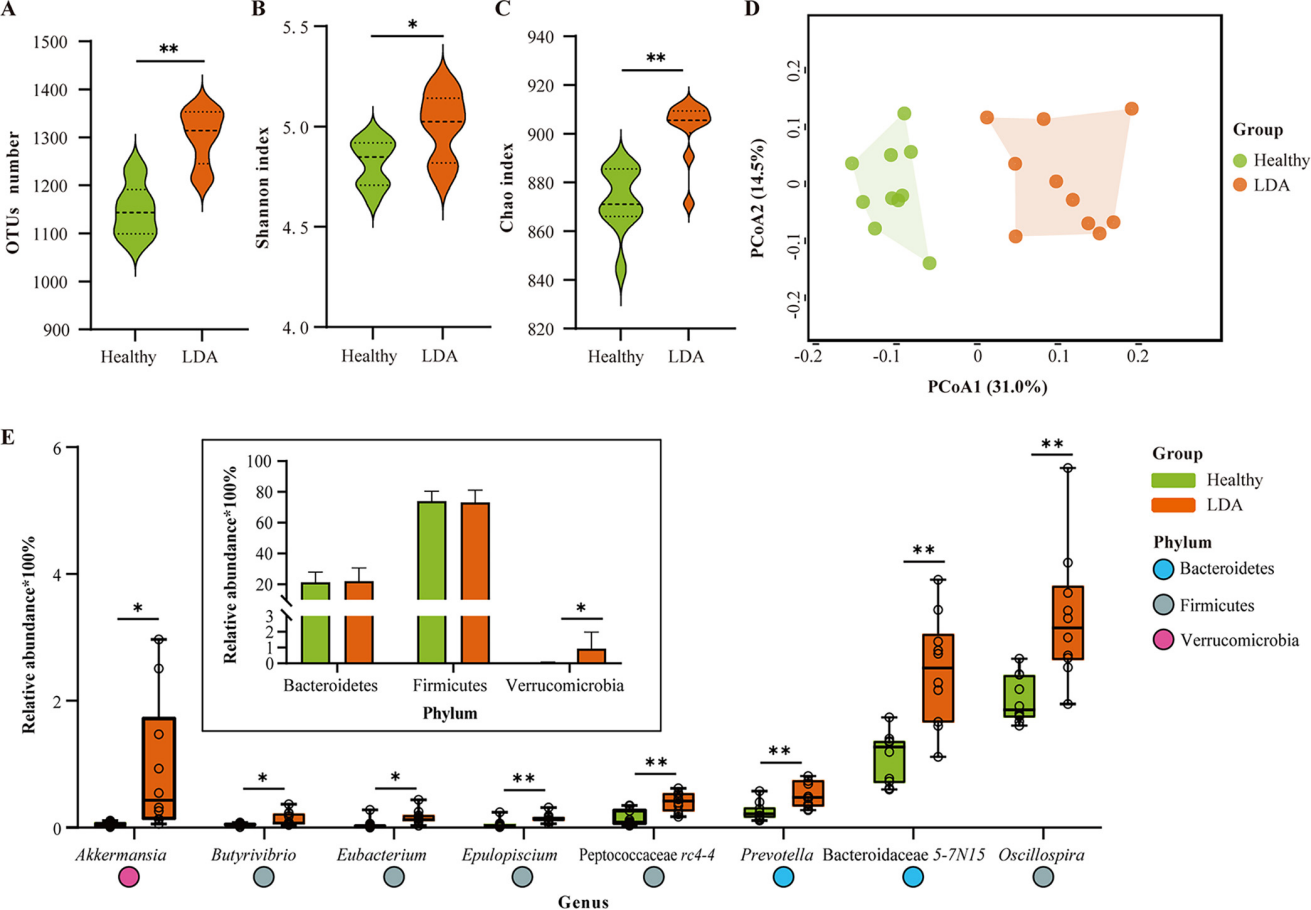
Fecal microbiome differ between healthy cows and left displaced abomasum (LDA) cows. (A) Variations in the numbers of operational taxonomic units (OTUs) in fecal samples. (B and C) Alpha diversity measured by the Shannon (B) and Chao1 (C) indexes were elevated in LDA cows compared with those of healthy cows. (D) Principal coordinates analysis based on the Bray-Curtis distance by permutational multivariate analysis of variance (PERMANOVA) showed different taxonomic compositions between the healthy and LDA groups. (E) Boxplot of gut microbiota at the genus level (only genera with relative abundance of >0.1% during LDA event and significance of *P < *0.05 are shown) between the healthy and LDA groups. Bar plot indicates that changes in phylum levels correspond to the targeted genera. The abundance of microbiota in the LDA group compared with that of the healthy group evaluated by analysis of variance. *, *P < *0.05; **, *P < *0.01.

Cows with LDA had higher OTU counts and alpha diversity compared to healthy cows (Fig. 1A to C), indicating that the gut microbiota richness increased due to the LDA event. Principal coordinate analysis showed that the fecal microbiomes between the LDA and healthy groups were clearly separated (Fig. 1D), and the heterogeneous community structure was significant (permutational analysis of variance [PERMANOVA] *P < *0.01), clearly separating the two groups.

Specifically, Firmicutes, Bacteroidetes, Tenericutes, Spirochaetes, Proteobacteria, and Actinobacteria were highly abundant in the feces of all lactating dairy cows (Table S3). Notably, the abundances of Verrucomicrobia, Fusobacteria, and Cyanobacteria were higher (*P < *0.05) in LDA cows than in healthy cows. Based on differential analysis of the gut microbiota at the genus level, cows with LDA had higher (*P < *0.05) abundances of 22 genera and lower (*P < *0.05) abundances of two genera compared with those of healthy cows (Table S4). We further focused on genera with a relative abundance of >0.1% in LDA events, including Peptococcaceae *rc4-4*, Bacteroidaceae *5-7N15*, *Oscillospira*, *Prevotella*, and *Epulopiscium*, which were markedly increased (*P < *0.01) in cows with LDA (Fig. 1E). Moreover, the abundance of *Akkermansia*, belonging to the Verrucomicrobia, was higher (*P < *0.05) in the LDA group than in healthy cows.

### Alterations of fecal metabolic profiles of cows with LDA.

**FIG 2:**
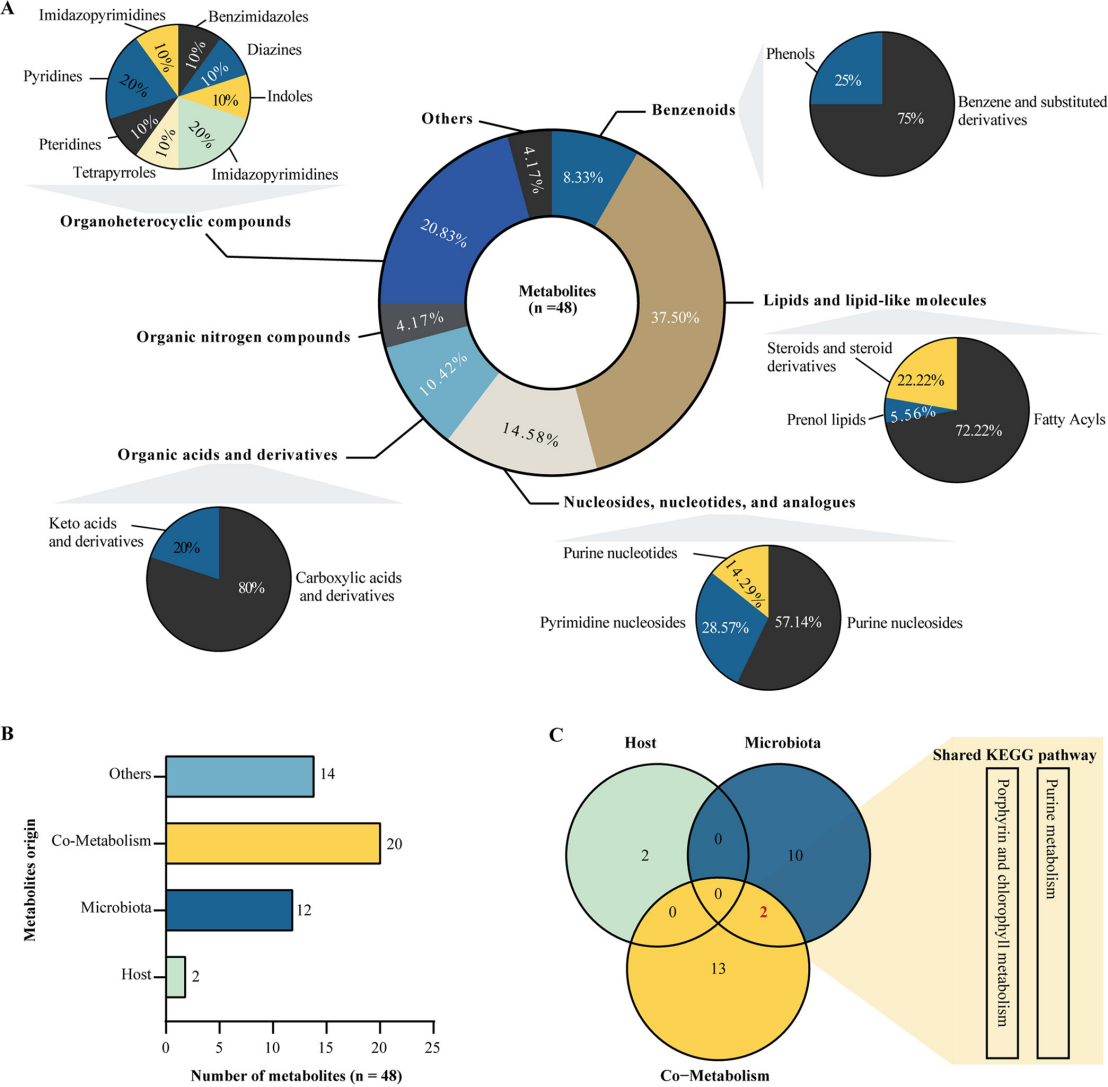
Fecal metabolites that differed between the healthy and LDA groups. (A) Hollow pie plot outlining the superclass proportion of 48 metabolites. Solid pie plot indicates the proportion of class metabolite in individual superclasses. (B) Bar plot indicating different origins of the differential metabolites, including host, microbiota, co-metabolism, and others (food-related, drug-related, and unknown). (C) Venn plot for KEGG pathway analysis of metabolites from different origins, including host, microbiota, and co-metabolism.

The principal component analysis and orthogonal partial least-squares discriminant analysis score plots showed that the fecal metabolome differed between the LDA and healthy groups (Fig. S1). A total of 48 metabolites differed between the healthy and LDA groups, including 20 with increased abundance and 28 with decreased abundance in cows with LDA (Table S5). These metabolites were classified as lipids (37.50%), nucleosides and nucleotides (14.58%), organic acids (10.42%), organoheterocyclic compounds (20.83%), and benzenoids (8.33%) (Fig. 2A). Moreover, 20 of the differential metabolites identified in the stool were derived from common metabolic processes between the host and gut microbiota (Fig. 2B), including linoleic acid, 4-pyridoxic acid, cytidine, citrulline, and cholic acid (Table S5). We also identified 12 differential metabolites specifically derived from the gut microbiota (Fig. 2B), including salicylic acid, glycodeoxycholic acid, and 2-methylbenzoic acid. Kyoto Encyclopedia of Genes and Genomes (KEGG) pathway analysis indicated two shared metabolic pathways between the microbiota and co-metabolism with the host (Fig. 2C), including purine metabolism and porphyrin-chlorophyll metabolism (Table S6).

### Associations of metabolites and fecal microbiota in LDA.

**FIG 3:**
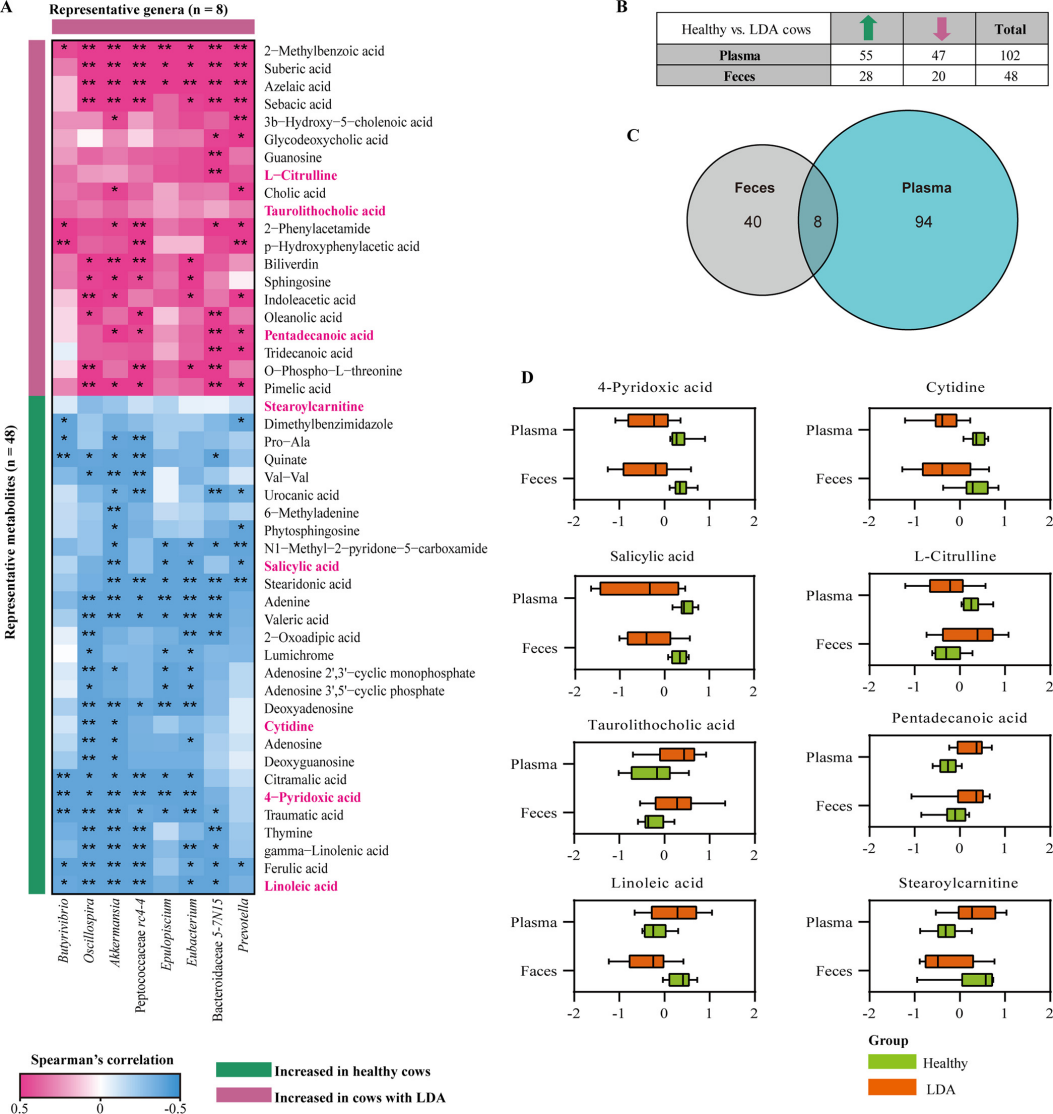
Associations of LDA-related genera and metabolites. (A) Heatmap depicting relationships between the gut microbiota and fecal metabolites which differed between healthy and LDA cows based on Spearman’s rank correlation analysis (*n* = 20). *, *P < *0.05; **, *P < *0.01. (B) Plasma and fecal metabolites which differed between the healthy and LDA groups. The numbers of significantly (*P < *0.05) increased and decreased metabolites in the LDA group compared with the healthy group are shown based on fold-change analyses in the plasma and feces, respectively. (C) Venn diagram outlining the shared metabolites in plasma and fecal samples. (D) Boxplots showing relative abundance of shared metabolites in plasma and fecal samples between the LDA and healthy groups. Relative abundances of metabolites are visualized after log_10_ transformation and scaling processing.

To identify correlations between metabolites and changes in the gut microbiome associated with LDA, we performed a Spearman’s rank correlation test. The abundances of *Akkermansia*, *Oscillospira*, and Peptococcaceae *rc4-4* were strongly correlated (*P < *0.01) with most of the metabolites identified in the feces (Fig. 3A). In our previous study, we identified 102 metabolites in the plasma which differed between LDA and healthy dairy cows (8). Therefore, we integrated the plasma metabolome with the fecal metabolome (Fig. 3B), and eight shared metabolites were screened between the fecal and plasma specimens, including citrulline, cytidine, linoleic acid, stearoylcarnitine, salicylic acid, 4-pyridoxic acid, taurolithocholic acid, and pentadecanoic acid (Fig. 3C and D). In comparison with those in healthy cows, the levels of cytidine and 4-pyridoxic acid, belonging to the co-metabolism category, were decreased (*P < *0.05) in the LDA group (Fig. 3D and Table S5). The level of salicylic acid produced by microbiota in dairy cows markedly decreased (*P < *0.01) during the LDA event. Linoleic acid and stearoylcarnitine levels of fecal samples were lower (*P < *0.05) in the LDA group than in the healthy group. By contrast, the plasma levels of linoleic acid and stearoylcarnitine were increased (*P < *0.05) in the LDA group.

We observed that the abundances of *Akkermansia* and *Oscillospira* were negatively correlated with levels of 4-pyridoxic acid (*r* = −0.68, *P = *0.001 and *r* = −0.50, *P = *0.028, respectively) and cytidine (*r* = −0.51, *P = *0.023 and *r* = −0.60, *P = *0.006, respectively) in the feces (Fig. 3A). The level of fecal salicylic acid was negatively correlated with the abundances of *Akkermansia* (*r* = −0.66, *P = *0.002) and *Prevotella* (*r* = −0.48, *P = *0.032). Of note, compared with the alterations in feces, the abundances of *Oscillospira* and Peptococcaceae *rc4-4* were positively correlated with linoleic acid (*r *=* *0.45, *P = *0.049 and *r *=* *0.55, *P = *0.013, respectively) and stearoylcarnitine (*r *=* *0.50, *P = *0.025 and *r *=* *0.60, *P = *0.006, respectively) levels in plasma (Fig. S2).

### Associations between omics data and clinical parameters.

**FIG 4:**
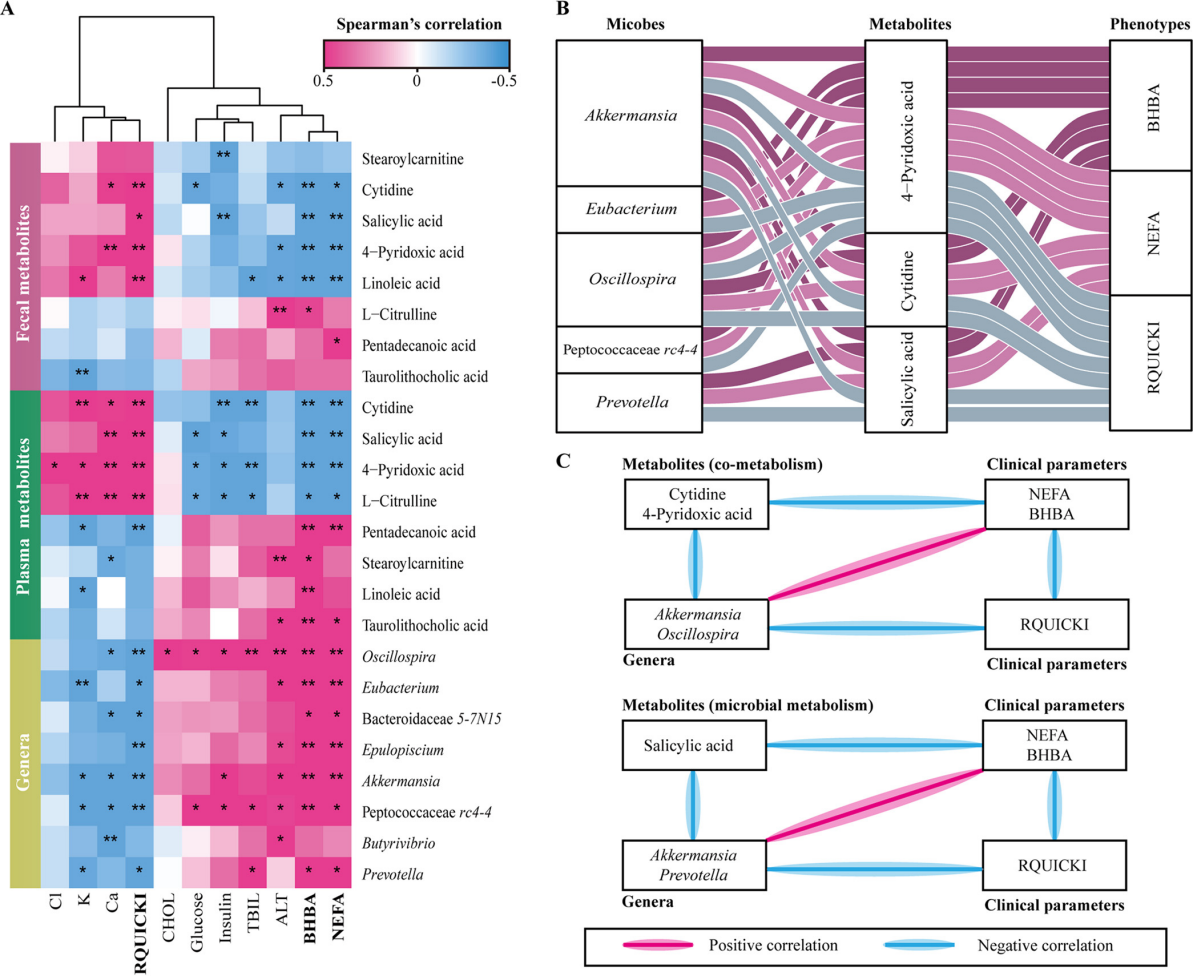
Associations of metabolites and gut microbiota with metabolic makers. (A) Heatmap depicting gut microbiota and shared metabolites compared with metabolic markers (only significant variables of *P < *0.05 are shown) using Spearman’s rank correlation analysis. Correlation and significance are indicated according to the legend. *, *P < *0.05; **, *P < *0.01. (B) Sankey plot outlining the relationships among the three shared metabolites (cytidine, 4-pridoxic acid, and salicylic acid) in the plasma and fecal samples linked to the gut microbiota and clinical phenotype, based on integration with the data shown in Fig. 3A and Fig. S2. (C) Flow plot outlining the relationships among metabolites, key gut microbial genera, and clinical parameters based on integration with the data shown in Fig. S3. Cytidine and 4-pridoxic acid participate in the pyrimidine metabolism (map00240) and pyridoxal metabolism (map00750) pathways, respectively.

We found that clinical markers differed markedly between the LDA and healthy groups, including NEFA, BHBA, glucose, insulin, cholesterol, alanine aminotransferase, total bilirubin, free calcium, chloride, and potassium levels, as well as the revised quantitative insulin sensitivity check index (RQUICKI), in serum (Table 1). To further investigate the relationships between gut microbiota, metabolites, and clinical markers, we focused on eight differential genera and eight shared metabolites that correlated with clinical variables using Spearman’s rank correlation test. We observed that NEFA, BHBA, and RQUICKI were critical variables associated with the LDA event (Fig. 4A), in which the NEFA and BHBA levels were negatively correlated with RQUICKI (*r* = −0.89, *P < *0.001 and *r* = −0.80, *P < *0.001, respectively; Fig. S3). The increases in circulating NEFA and BHBA concentrations were strongly positively correlated with increases in the abundance of *Akkermansia* (*r *=* *0.60, *P = *0.005 and *r *=* *0.70, *P < *0.001, respectively), *Oscillospira* (*r *=* *0.70, *P < *0.001 and *r *=* *0.81, *P < *0.001, respectively), Peptococcaceae *rc4-4* (*r *=* *0.54, *P = *0.013 and *r *=* *0.64, *P = *0.002, respectively), and *Prevotella* (*r *=* *0.50, *P = *0.026 and *r *=* *0.51, *P = *0.022, respectively; Fig. 4A). In addition, the decrease in RQUICKI was negatively correlated with increases in the abundance of *Akkermansia* (*r* = −0.64, *P = *0.003), *Oscillospira* (*r* = −0.70, *P < *0.001), Peptococcaceae *rc4-4* (*r* = −0.63, *P = *0.003), and *Prevotella* (*r* = −0.49, *P = *0.029). Notably, the changes in 4-pridoxic acid, cytidine, and salicylic acid levels in the feces and plasma were consistent (Fig. 2D), and these metabolites were negatively correlated (*r* < −0.4, *P < *0.05) with NEFA and BHBA concentrations (Fig. 4A). Moreover, these three key metabolites were linked to the gut microbiota and metabolic phenotype (Fig. 4B). Subsequently, we integrated the correlations between metabolites and microbiota, which screened out three key metabolites and three genera (Fig. 4C). KEGG pathway analyses indicated that 4-pridoxic acid and cytidine participate in pyridoxal metabolism and pyrimidine metabolism, respectively (Table S6).

**TABLE 1:** Production events and metabolic markers of healthy cows and cows with left displaced abomasum^*a*^

Event or marker	Healthy cows (*n* = 10)	LDA cows (*n* = 10)
Production event^*b*^		
Parity	2.00 ± 0.71	1.89 ± 0.79
Days in milk (d)	18.22 ± 1.99	17.78 ± 3.38
Milk yield (kg)	29.71 ± 2.38	16.81 ± 1.89^*c*^
Metabolic marker^*d*^		
NEFA (mmol/L)	0.35 ± 0.04	0.89 ± 0.11^*c*^
BHBA (mmol/L)	0.87 ± 0.08	1.90 ± 0.27^*c*^
Glucose (mmol/L)	2.56 ± 0.31	3.29 ± 0.89^*e*^
Insulin (μU/mL)	7.95 ± 0.78	8.9 ± 0.90^*e*^
RQUICKI	0.48 ± 0.02	0.39 ± 0.02^*c*^
TG (mmol/L)	0.18 ± 0.02	0.21 ± 0.07
ALT (U/L)	23.70 ± 3.95	32.90 ± 11.45^*e*^
CHOL (mmol/L)	3.71 ± 0.65	4.48 ± 0.92^*e*^
TP (g/L)	77.60 ± 4.90	84.00 ± 10.67
ALB (g/L)	28.90 ± 1.66	28.60 ± 3.47
GLOB (g/L)	48.80 ± 4.37	55.40 ± 10.02
TBIL (μmol/L)	3.30 ± 1.34	5.40 ± 1.43^*c*^
Ca (mmol/L)	2.41 ± 0.08	2.12 ± 0.22^*c*^
K (mmol/L)	4.68 ± 0.29	3.90 ± 0.60^*c*^
Cl (mmol/L)	105.70 ± 2.98	101.80 ± 4.61^*e*^

a LDA, left displaced abomasum; NEFA, non-esterified fatty acid; BHBA, β-hydroxybutyric acid; RQUICKI, revised quantitative insulin sensitivity check index; TG, triglyceride; ALT, alanine aminotransferase; CHOL, cholesterol; TP, total protein; ALB, albumin; GLOB, globulin; total bilirubin, TBIL.

b Production data were obtained from our previous report (8), including parity, days in milk, and milk yield.

c *P *<* *0.01 compared to healthy cows using two-tailed Student’s *t* test.

d Data of NEFA and BHBA as metabolic markers were obtained from our previous study (8). Serum biochemistry was determined using automatic biochemical analyzer (Catalyst One, IDEXX, Westbrook, USA), including glucose, TG, ALT, CHOL, TP, ALB, GLOB, and TBIL. Serum electrolytes were determined using a VetStat Analyzer (IDEXX, Westbrook, USA), including free calcium (Ca), potassium (K), and chloride (Cl). Serum insulin levels were determined using a commercial enzyme-linked immunoassay kit.

e *P *<* *0.05 compared to healthy cows using two-tailed Student’s *t* test.

To determine LDA-specific biomarkers, we performed multiple logistic regression and receiver operating characteristic (ROC) analyses using the identified key metabolites associated with LDA, including 4-pyridoxic acid, salicylic acid, and cytidine. The area under the ROC curve value was 0.91 and 0.98 for the feces and plasma, respectively (Fig. S4). In our dairy cow study cohort, 9 of the 10 healthy cows were successfully predicted and 9 of the 10 cows with LDA were successfully predicted.

### Alterations of plasma amino acids in cows with LDA.

**FIG 5:**
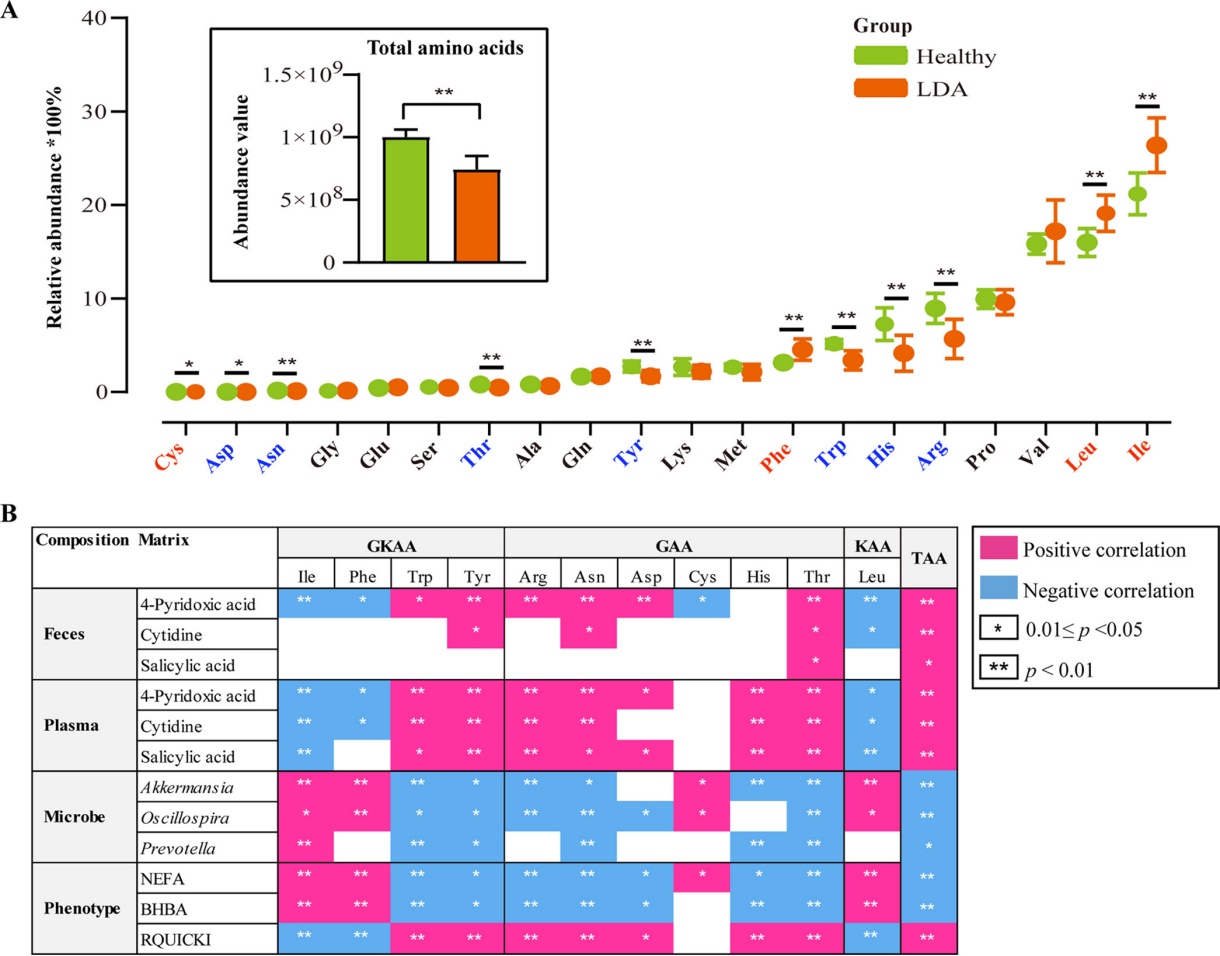
Changes in circulating amino acids in dairy cows with LDA. (A) Upper left bar plot outlines the abundance of plasma total amino acids in healthy cows and cows with LDA. Scatterplot reflects the relative mean abundance of plasma amino acids between healthy cows and cows with LDA. Relative abundance was calculated by dividing the levels of individual amino acids by the level of total amino acids. Red (blue) text indicates the relative abundances of amino acids which are elevated (decreased) in LDA cows compared with that in healthy cows. (B) Complex variable panel results for microbiota, metabolites, and phenotype compared with the levels of 11 amino acids (only significantly different amino acids of *P < *0.05 are shown) across the top, categorized according to glycogenic (GAA), ketogenic (KAA), glycogenic and ketogenic (GKAA), and total (TAA) amino acids. Correlation and significance are indicated as described in the legend.

We performed targeted metabolomics to analyze the pool and composition of amino acids. The plasma level of total amino acids was markedly decreased in the LDA group compared with the healthy group, indicating that the size of the amino acid pool waned in the LDA event (Fig. 5A). The relative abundances of BCAA, including leucine and isoleucine, were markedly increased (*P < *0.01) in the LDA group compared with that in the healthy group (Fig. 5A). In contrast, the abundances of tyrosine, tryptophan, and histidine decreased (*P < *0.01) in the LDA group. Based on the key metabolic factors and associated microbes linked to the LDA event screened out from the correlation analysis described above (Fig. 4C), we further analyzed the correlations between various amino acids, metabolic factors, and microbes. The decrease in circulating total amino acid levels was strongly negatively correlated with an increase in the abundance of microbes, including *Akkermansia* (*r* = −0.68, *P = *0.001) and *Oscillospira* (*r* = −0.68, *P = *0.001; Fig. 5B). By contrast, total amino acid levels were positively correlated (*r *>* *0.5, *P < *0.02) with the abundances of the key metabolites 4-pyridoxic acid, cytidine, and salicylic acid in the feces and plasma. The decrease in fecal 4-pyridoxic acid levels was positively correlated with decreases in most glycogenic amino acids (*r *>* *0.5, *P < *0.02) and negatively correlated with the increase in leucine (*r* = −0.64, *P = *0.004). Moreover, the increased levels of leucine and isoleucine, which belong to the ketogenic amino acid group, were positively correlated with increases in the abundance of *Akkermansia* (*r *=* *0.66, *P = *0.002 and *r *=* *0.61, *P = *0.005, respectively) and *Oscillospira* (*r *=* *0.56, *P = *0.011 and *r *=* *0.49, *P = *0.029, respectively). Notably, the increase in NEFA and BHBA concentrations was strongly positively correlated with an increase in the levels of BCAA, including leucine (*r *=* *0.59, *P = *0.006 and *r *=* *0.61, *P = *0.004, respectively) and isoleucine asparagine (*r *=* *0.62, *P = *0.004 and *r *=* *0.65, *P = *0.002, respectively). However, the decrease in RQUICKI was strongly negatively correlated with an increase in leucine (*r* = −0.82, *P < *0.001) and isoleucine levels (*r* = −0.72, *P < *0.001).

### Metabolic pathway and network of LDA.

**FIG 6:**
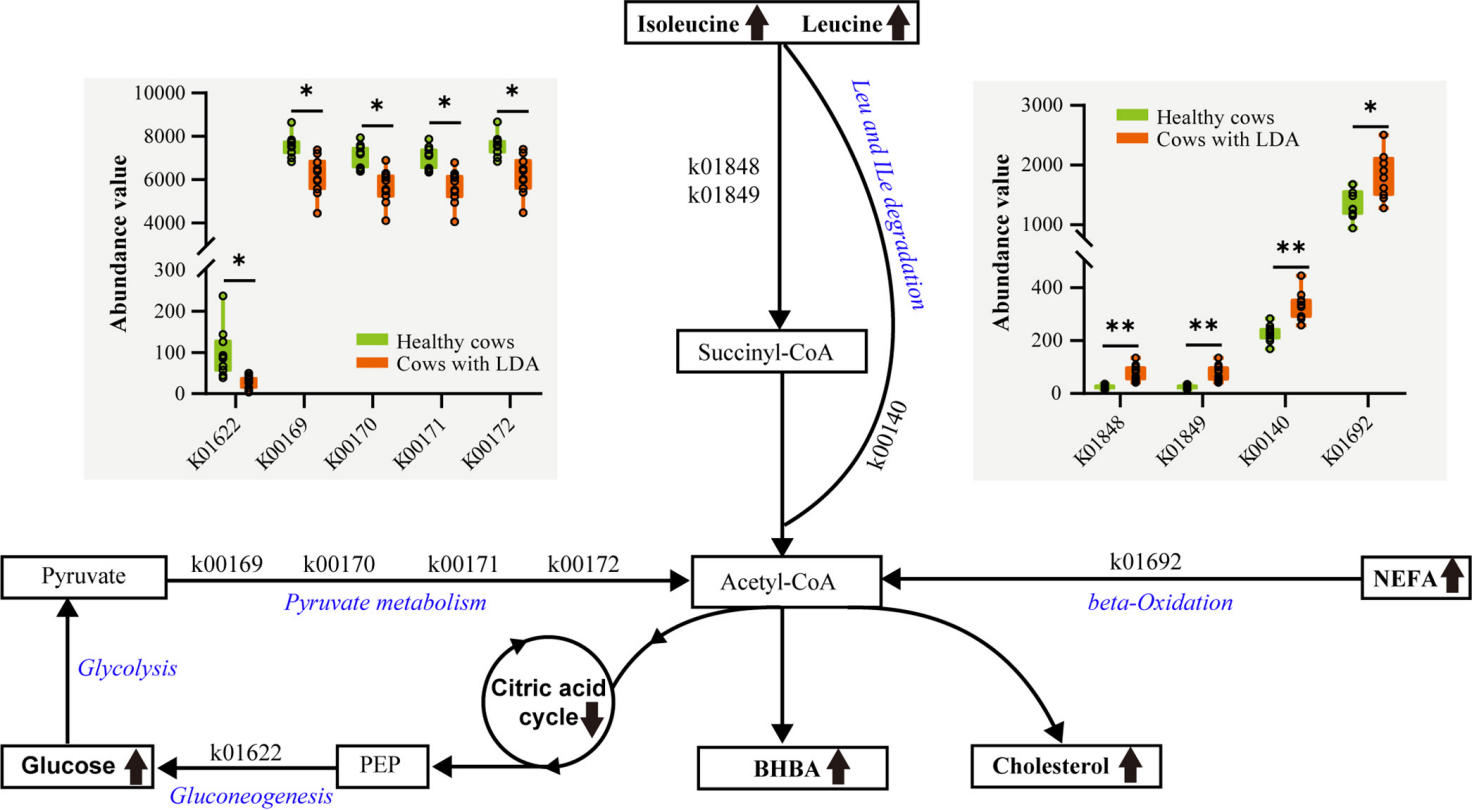
Prediction of genes based on KEGG orthologs (KO) associated with plasma levels of leucine and isoleucine, and genes involved in leucine-isoleucine degradation and fatty acid beta-oxidation. Boxplots indicate differential enrichment of KOs in healthy cows and cows with LDA. k00140, malonate-semialdehyde dehydrogenase; k01692, enoyl-CoA hydratase; k01848, methylmalonyl-CoA mutase (N-terminal domain); k01849, methylmalonyl-CoA mutase (C-terminal domain); k00169, pyruvate ferredoxin oxidoreductase alpha subunit; k00170, pyruvate ferredoxin oxidoreductase beta subunit; k00171, pyruvate ferredoxin oxidoreductase delta subunit; k00172, pyruvate ferredoxin oxidoreductase gamma subunit; k01622, fructose-1. NEFA, non-esterified fatty acid; BHBA, beta-hydroxybutyric acid; PEP, phosphoenolpyruvic acid. An upward-pointing arrow indicates that the metabolite level was increased in the LDA group compared with that in the healthy group, whereas a downward-pointing arrow indicates that the metabolite level was decreased in the LDA group, such as the change in citric acid level from that in our previous report (8). The enzymatic reactions involved are shown in Table S8. *, *P < *0.05; **, *P < *0.01.

We identified 3,697 KEGG orthologs (KOs) in the fecal samples, 203 of which differed in abundance between LDA and healthy cows (adjusted *P < *0.05; Table S7). Spearman’s correlation analysis showed that the relative abundances of leucine and isoleucine were strongly correlated with NEFA and BHBA concentrations (Fig. 5B), indicating that ketogenic amino acid metabolism increased in the LDA event. Genes encoding enzymes produced by gut microbes were found to mediate these ketogenic relationships (Fig. 6, Table S8), including methylmalonyl-CoA mutase (k01848 and k01849, conversion of propanoyl-CoA to succinyl-CoA), alonate-semialdehyde dehydrogenase (k00140, conversion of propanoyl-CoA to acetyl-CoA), and enoyl-CoA hydratase (k01692, β-oxidation). Notably, glycolysis/gluconeogenesis and the citric acid cycle were downregulated in the LDA event. Moreover, decreased levels of tryptophan and tyrosine, which are representative glycogenic and ketogenic amino acids, respectively, were strongly and positively correlated with decreased levels of 4-pyridoxic acid (*r *>* *0.55, *P < *0.01) in the feces and plasma (Fig. 5B).

**FIG 7:**
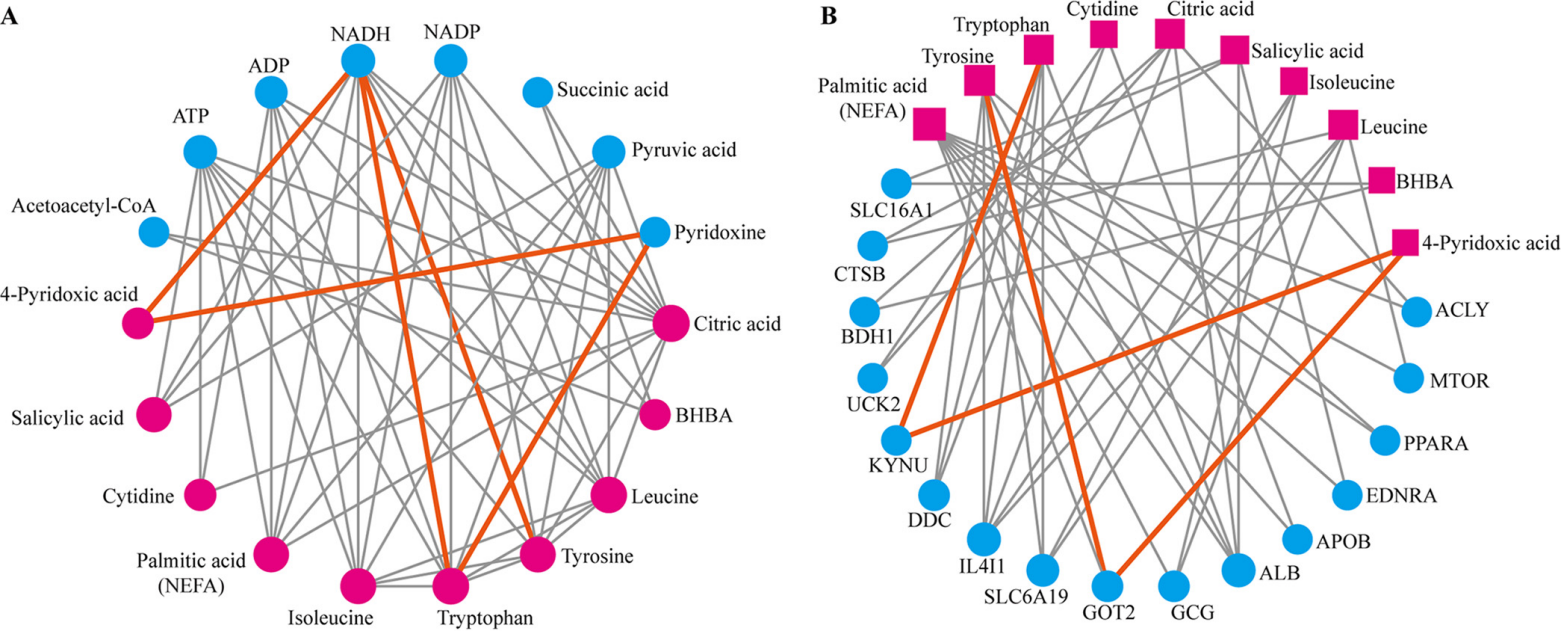
Metabolic network of key metabolites. (A) Metabolite-metabolite interaction network of target metabolites. Target metabolites are presented as red nodes and associated metabolites are presented as blue nodes. NADH, reduced form of nicotinamide-adenine dinucleotide. (B) Metabolite-gene interaction network of the target metabolites. Target metabolites are presented as red nodes and associated genes are presented as blue nodes. GOT2, glutamic-oxaloacetic transaminase 2; KYNU, kynureninase; SLC16A1, solute carrier family 16 member 1; CTSB, cathepsin B; BDH1, 3-hydroxybutyrate dehydrogenase 1; UCK2, uridine-cytidine kinase 2; DOC, dopa decarboxylase; IL-4I1, interleukin 4-induced 1; SLC6A19, solute carrier family 6 member 19; GOT2, glutamic-oxaloacetic transaminase 2; GCG, glucagon; ALB, albumin; APOB, apolipoprotein B; EDNRA, endothelin receptor type A; PPAR, peroxisome proliferator activated receptor alpha; MTOR, mechanistic target of rapamycin kinase; ACLY, ATP citrate lyase.

Since we previously reported that tryptophan and tyrosine metabolism were negatively correlated with NEFA and BHBA levels in dairy cows under metabolic stress (20), we constructed metabolite-metabolite and metabolite-gene interaction networks to investigate whether 4-pyridoxic acid, salicylic acid, and cytidine are involved in the ketogenic reaction. Interestingly, pyridoxine was identified as a key metabolite linking 4-pyridoxic acid to tryptophan in the network (Fig. 7A). Pyridoxal metabolism was also associated with NEFA, BHBA, and RQUICKI (Fig. 4C). Additionally, kynureninase was identified as a critical gene mediating the association between 4-pyridoxic acid and tryptophan (Fig. 7B), whereas the glutamic-oxaloacetic transaminase-2 was identified as mediating the link between 4-pyridoxic acid and tyrosine.

## DISCUSSION

Dairy cows are at increased risk of developing metabolic disorders as a result of LDA events (7, 16); however, the associated mechanisms contributing to metabolic changes in LDA remain unclear. In this study, we used integrated multi-omics data to analyze the correlations among gut microbiota, metabolites, and host phenotypes in dairy cows with LDA. The results clearly demonstrate a state of gut microbiota dysbiosis associated with an amino acid deficit in dairy cows with LDA. Changes in the gut microbiota composition were strongly correlated with ketogenesis and glucose metabolism in the context of LDA. Furthermore, key metabolites which link microbiota to phenotypes in cows with LDA were identified, providing novel mechanistic insights into disease pathogenesis.

Gastrointestinal microbiota dysbiosis is closely related to the development of metabolic diseases such as obesity and diabetes (17). The rumen microbiota composition has been associated with production and health in dairy cows (21, 22). Hu et al. (23) reported that dysbiosis of the rumen microbiota contributes to the development of diseases in dairy cows. However, knowledge of the contribution of the lower gut microbiota to the health and production of dairy cows is limited (24). In the present study, we found that gut microbiota richness of dairy cows increased during LDA events, and circulation amino acid pool deficits in LDA events indicated that dairy cows with LDA are characterized by low dry matter intake. Previous studies have explained that intermittent fasting increased the richness and diversity of gut microbiota, which are associated with the balance of energy demand during low-energy intake status (25, 26). Notably, a recent study revealed that the microbiota structure was markedly different between the rumen and gut in dairy cows during early lactation, with Firmicutes and Bacteroidetes being the most abundant taxa in the gut (27). We found no alteration in the abundance of Firmicutes and Bacteroidetes in the gut associated with LDA during lactation, whereas the abundance of Fusobacteria was markedly higher in cows with LDA than in healthy cows. An increased abundance of Fusobacteria has also been reported in the context of metabolic disorders and inflammation (28). Additionally, we found that the abundance of Saccharibacteria (TM7) was decreased in LDA cows, which has been reported to supplement the contribution of Saccharibacteria (TM7) to reduced inflammation by modulating the pathogenicity of the host bacteria (29). Previous studies have also suggested that LDA increases the levels of inflammatory biomarkers, highlighting an inflammation challenge in dairy cows during LDA events (30). Thus, the associations of the observed changes in Fusobacteria and Saccharibacteria (TM7) in the gut of cows with LDA warrant further study.

We also observed that the abundances of *Oscillospira*, Bacteroidaceae *5-7N15*, *Prevotella*, *Akkermansia*, *Butyrivibrio*, Peptococcaceae *rc4-4*, *Eubacterium*, and *Epulopiscium* were higher in cows with LDA than in healthy cows. Intriguingly, changes in the abundances of *Oscillospira* (31), *Akkermansia* (32), *Prevotella* (33), and Peptococcaceae *rc4-4* (34) have also recently been recognized as important factors in the development of metabolic diseases. *Akkermansia* belongs to the phylum Verrucomicrobia, whose abundance has been reported to increase during low-grade inflammation status (35). In addition, a previous study showed that an increased abundance of *Prevotella* induces low-grade inflammation by mediating the T helper 17 cell immune response (36). Low-grade inflammation has been linked to enhanced lipid mobilization in dairy cows and has also been implicated as a contributor to metabolic diseases during the lactation period, including ketosis and LDA (37). We further observed positive correlations in the abundances of *Akkermansia* and *Prevotella* with NEFA and BHBA levels, suggesting that *Akkermansia* and *Prevotella* contribute to intensifying lipolysis in cows with LDA. Several studies have suggested that cows with LDA have enhanced lipolysis and ketogenesis (7, 8, 13). Notably, Bacteroidaceae, *Oscillospira*, and *Butyrivibrio* are important microbes that produce butyrate (31, 38). In addition to being an important short-chain fatty acid for maintaining epithelial integrity, butyrate is also used as a substrate to synthesize ketone bodies and cholesterol in the liver (39). However, whether butyrate from gut microbiota catabolism plays a dual role in development of LDA remains to be further investigated. We speculate that the observed gut microbiota dysbiosis is associated with the high circulating NEFA and BHBA concentrations in dairy cows with LDA.

Recently, Zierer et al. (40) demonstrated that the fecal metabolome can be used to understand the function of gut microbial activity and the associations of fecal metabolites with host metabolism. To explore the correlative mechanism between gut microbiota and the metabolic phenotype in cows with LDA, we compared the fecal metabolome profiles between LDA and healthy dairy cows. The differential metabolites between the two groups were involved mainly in co-metabolism, with 4-pyridoxic acid and cytidine identified as key metabolites. 4-Pyridoxic acid is the main catabolite of pyridoxal, which is associated with systemic inflammation (41); thus, 4-pyridoxic acid is widely used as a functional biomarker to evaluate the pyridoxal status in the body (42). In the present study, the levels of 4-pyridoxic acid in the feces and plasma were markedly lower in LDA cows than in healthy cows. We also observed that 4-pyridoxic acid was strongly positively correlated with tryptophan and mediated the link between the gut microbiota and clinical phenotype. Guo et al. (16) suggested pyridoxal deficiency as an evident characteristic in cows with LDA, along with a concurrent decrease in tryptophan metabolism. Pyridoxal deficiency has been widely reported to be associated with inflammatory or metabolic diseases (42). Mascolo et al. (43) suggested that pyridoxal participates in regulating the inflammatory response and oxidative stress by altering kynureninase activity in the tryptophan metabolism pathway. In addition, we found that the abundance of salicylic acid, which is derived from the catabolism of gut microbiota, decreased during LDA events. Zhang et al. (44) reported that salicylic acid negatively modulates the abundance of *Prevotella*, further suppressing the inflammation response. Of note, 4-pyridoxic acid, cytidine, and salicylic acid were regarded as prediction factors of LDA in this study. However, one limitation of this study is that the small sample size may increase the false positive rate in the ROC curve.

Conversely, we observed increases in other gut microbiota catabolites in cows with LDA compared with that in healthy cows, such as glycodeoxycholic acid and taurolithocholic acid (TLCA), which belong to the secondary bile acids (SBA) group. Taurochenodeoxycholic acid (TCDCA) is transformed into lithocholic acid (LCA) and TLCA by the gut microbiota via an alternative bile acid synthetic pathway (45). LCA and TLCA stimulate glucagon-like peptide-1 (GLP-1) secretion to regulate glucose metabolism by activating Takeda G protein-coupled receptor 5 (TGR5) (46). GLP-1 is involved in the glucose-dependent stimulation of insulin secretion, which contributes to increased insulin levels in cows with LDA. Pravettoni et al. (9) demonstrated that high glucose levels and low insulin sensitivity were characteristics of LDA, similar to ketosis. Although SBA deficiency promotes the development of intestinal inflammation, a high SBA level associated with metabolic diseases has also been reported (46). Using a fecal transplantation experiment, de Groot et al. (47) found that fecal LCA and plasma TLCA levels were markedly increased in metabolic syndrome. Indeed, the plasma TCDCA and TLCA levels were increased in LDA cows in the present study, as reported in our previous study (8). Collectively, these findings suggest that the gut microbiota plays an important role in LDA development.

Accumulating evidence suggests that dairy cows experience an excessive nutrient or amino acid deficit in cases of metabolic disease (8, 48). Consistently, we observed that the size of the amino acid pool decreased in LDA cows compared with that in healthy cows and that the composition of the amino acid pool was also altered. Notably, the relative proportion of BCAA was increased in cows with LDA and positively correlated with the levels of BHBA and NEFA, such as leucine and isoleucine. BCAA can be converted to acetyl-CoA or succinyl-CoA, which participate in the tricarboxylic acid cycle during a state of nutrient deficiency, and then acetyl-CoA produces BHBA (49). Interestingly, we found that ketogenic genes were upregulated in LDA cows, while tricarboxylic acid cycle and glycolysis/gluconeogenesis-related genes were downregulated in LDA. Additionally, BCAA is synthesized by the gut microbiota and maintains homeostasis by regulating glucose metabolism, lipid metabolism, and insulin resistance (18). Yoon (50) suggested that high plasma BCAA levels contribute to insulin resistance by activating the mammalian target of rapamycin complex-1. Most metabolic diseases in dairy cows are closely related to insulin resistance, such as ketosis, fatty liver, and LDA (9, 51, 52). However, previous studies have suggested that abnormal metabolism of aromatic amino acids, including tryptophan and tyrosine, is a risk factor for inducing insulin resistance (53, 54). We observed that the gut microbe was negatively correlated with tryptophan and tyrosine metabolism. Taken together, these findings suggest that the gut microbe is involved in the regulation of lipolysis and insulin resistance in LDA by modulating the amino acid composition, which requires further research.

In conclusion, to our knowledge, this is the first study to characterize the correlation between gut microbes and metabolic phenotypes in dairy cows with LDA by leveraging multi-omics data. Key metabolites were identified that were closely related to gut microbiota dysbiosis, and lipolysis was enhanced in conjunction with an amino acid deficit in LDA cows, including 4-pyridoxic acid, cytidine, and salicylic acid. In contrast, changes in SBA and BCAA metabolism in the gut microbiota may contribute to the promotion of insulin resistance. One of the main limitations of this study is that our findings revealed functional links between the microbiome, metabolome, and disease phenotype, but could not define causality. Therefore, we suggest future evaluation of the correlation between the gut microbiota and metabolism prior to the onset of the disease, which could further help explain the underlying cause of LDA and suggest potential new prevention options.

## MATERIALS AND METHODS

### Animals and sample collection.

Animals were treated and samples were collected in strict accordance with the Guidelines for the Care and Use of Laboratory Animals of China. All procedures were approved by the Institutional Animal Care and Use Committee of Sichuan Agricultural University (no. DYY-2018203039). The experiment was performed at a modern dairy farm in Sichuan Province, China from December 2019 to March 2020, with approximately 1,200 lactation Holstein dairy cows, 350 dairy heifers, and 450 dry cows. All cows were transferred to fresh barns after calving. Lactation dairy cows were housed in free-stall barns and had free access to freshwater. The cows were milked daily at 0630, 1200, and 1930 h. A total mixed ration (TMR) diet was provided three times daily (ingredients and chemical composition of the TMR diet are shown in Table S1). After the first daily milking, a veterinarian monitored for and diagnosed diseases in the postpartum dairy cows using previously described evaluation criteria (4).

The average monthly incidence of LDA in total herds was 1.3% during the experimental period, 300 postpartum dairy cows were included in clinical health monitoring, and 26 cows were diagnosed with LDA. The clinical criteria used to determine a healthy status and an LDA diagnosis have been described in our previous study (8). LDA diagnosis and sample collection were performed prior to feeding. Blood and stool samples were collected on the day of LDA diagnosis from both the LDA cows and healthy cows. Serum and plasma (heparin sodium as an anticoagulant) samples were collected and centrifuged at 1,500 × *g* for 10 min at 25°C. All samples were stored at −80°C until analysis. Only cows with a single LDA event were included in the study cohort. Finally, we selected 10 healthy postpartum Holstein dairy cows and 10 LDA dairy cows with similar parity, age, and days in milk. The production data were collected, and the serum metabolic markers assessed in this study are listed in Table 1.

### Fecal microbiome analysis by 16S rDNA amplicon sequencing.

DNA was extracted from the stool samples using the E.Z.N.A. Stool DNA kit (no. D4015; Omega Bio-Tek, Norcross, GA, USA). DNA concentration and purity were determined using a 1% agarose gel. According to the concentration, DNA was diluted to 1 ng/μL using sterile water. Amplification of the 16S rDNA V3-V4 region was performed by PCR (Bio-Rad, Hercules, CA, USA) as described previously (55). The 16S rDNA was sequenced on the MiSeq platform (HiSeq2500; Illumina, San Diego, CA, USA). After raw sequences were filtered, paired-end reads were merged to tags by fast-length adjustment of short reads (FLASH, v1.2.8). The tags were clustered into operational taxonomic units (OTUs) with a 97% threshold using UPARSE pipeline (56). Chimeras were filtered out using UCHIME (v4.2.40). Each OTU representative sequence was taxonomically classified using Ribosomal Database Project Classifier trained on the Greengenes database by QIIME (v1.8.0), based on a 0.7 confidence threshold. The OTU tables were filtered to remove low-quality features based on interquartile range, and then the data were normalized by total sum scaling. After data pretreatment, alpha and beta diversities were calculated using the MicrobiomeAnalyst platform (https://www.microbiomeanalyst.ca) (57).

### Fecal metabolome profiling.

Stool samples (60 mg) were added to 1,000 μL of methanol:acetonitrile:water (4:4:1, vol/vol/vol) containing an isotopically labeled internal standard mixture. After vortexing for 60 s, the samples were sonicated twice using ultrasonic liquid processors (Scientz JY92-II, Ningbo, China) in an ice-water bath for 30 min. The samples were then incubated for 1 h at −20°C and centrifuged at 14,000 × *g* for 20 min at 4°C. The supernatants were subjected to ultra-high-performance liquid chromatography (UHPLC; 1290 Infinity II, Agilent Technologies, Santa Clara, CA, USA). The column temperature was set to 25°C and the flow rate was 0.3 mL/min. Samples were processed with the following mobile phase gradient: 0 to 0.5 min, 95% B (acetonitrile); 0.5 to 7 min, 95% to 65% B; 7 to 8 min, 65% to 40% B; 8 to 9 min, 40% B; 9 to 9.1 min, 40% to 95% B; 9.1 to 12 min, 95% B. Mobile phase A consisted of water with 25 mM ammonium acetate and 25 mM ammonium hydroxide. Triple time-of-flight was performed on a mass spectrometer (TOF 6600, AB SCIEX, Framingham, MA, USA) equipped with an electrospray ionization source operating in positive- and negative-ion modes. The source temperature was 600°C and the ion source gas and curtain gas pressures were 0.4137 MPa and 0.20685 MPa, respectively. The mass-to-charge ratio (*m/z*) ranges of the time-of-flight MS scan and tandem mass spectrometry ion scan were set to 60 to 1,000 Da and 25 to 1,000 Da, respectively. The MS/MS spectra were collected in information-dependent acquisition mode. The ion peak, retention time, and peak area were analyzed using XCMS software. The detailed approach used for compound identification of the metabolites is described in our previous report (58).

### Targeted plasma amino acids analyses.

The plasma samples were pretreated as previously described (58) and the supernatants were prepared using the UHPLC system (1290 Infinity II, Agilent Technologies). The column temperature was 40°C, the injection volume was 1 μL, and the flow rate was 0.25 mL/min. The mobile phase consisted of A (water with 25 mM ammonium formate and 0.08% formic acid) and B (acetonitrile with 0.1% formic acid). The mobile phase gradient was 0 to 12 min, 90% to 70% B; 12 to 18 min, 70% to 50% B; 18 to 25 min, 50% to 40% B; 25 to 30 min, 40% B; 30 to 30.1 min, 40% to 90% B; 30.1 to 37 min, 90% B. MS (QTRAP 5500, AB SCIEX, Framingham, MA, USA) was performed in positive-ion mode. The source temperature was 500°C, and the ion source gas and curtain gas pressures were 0.2758 MPa and 0.20685 MPa, respectively. Amino acid data were collected with a multiple reaction monitor, and the peak area and retention time were collected using MultiQuant software (v3.0, AB SCIEX). The targeted amino acids were identified using standard substances from Sigma-Aldrich (Saint Louis, MO, USA), as shown in Table S2.

### Statistical analyses.

To compare the variables between the healthy and LDA groups, univariate analyses were performed in R software (v 4.1.3), including fold change and a two-tailed Student’s *t* test. The fecal metabolic profile data were log_10_-transformed and scaled to multivariate analysis. Principal-component analysis and orthogonal partial least-squares discriminant analysis were performed using SIMCA-P software (v14.1.0, Umetrics, Umea, Sweden). A *P* value of less than 0.05 was considered significant. Differential metabolites were screened using variable importance in projection scores of >1 and *P < *0.05. The category and origin of the differential metabolites were analyzed using the MetOrigin platform (59).

To screen for key metabolites, the fecal microbiome and its metabolome were integrated with plasma metabolome data (MetaboLights repository, accession code MTBLS4126) obtained in our previous study (8). The Spearman’s rank correlation coefficient was used to analyze the associations between fecal microbiota and differential metabolites. Correlations between clinical variables and microbiota/metabolites were also assessed in both LDA and healthy cows. Correlation thresholds were set to Spearman’s |*r*| > 0.4 and *P* < 0.05. KEGG pathway and KO functional enrichment analyses were performed using the R platform. Network interaction analysis between metabolites and metabolites/genes was performed using MetaboAnalyst (https://www.metaboanalyst.ca) (60). To determine relevant clinical biomarkers of LDA, multiple logistic regression and receiver operating characteristic curve analyses of metabolites were performed using GraphPad software (v9.1.0, GraphPad, San Diego, CA, USA).

### Data availability.

Sequencing data are available on the NCBI Sequence Read Archive (SRA) database (https://www.ncbi.nlm.nih.gov/sra) under the study accession code PRJNA838477. The raw LC-MS/MS data files for the fecal metabolome are deposited at the MetaboLights database (http://www.ebi.ac.uk/metabolights) of the European Bioinformatics Institute under the code MTBLS2441.
