# Supplementary material for: Altered Fecal Microbiome and Correlations of the Metabolome with Plasma Metabolites in Dairy Cows with Left Displaced Abomasum
Source: Microbiol Spectr. 2022 Oct 12;10(6):e01972-22. doi: 10.1128/spectrum.01972-22 (PMC9769586; doi:10.1128/spectrum.01972-22)
Supplement: Supplemental file 1 — Fig. S1 to S4;Tables S1 to S4, S6, and S8. Download spectrum.01972-22-s0001.pdf, PDF file, 0.7 MB [file spectrum.01972-22-s0001.pdf]

Supplementary information

Altered Fecal Microbiome and Correlations of the Metabolome with Plasma Metabolites in Dairy Cows with Left Displaced Abomasum

Zhengzhong Luo, [a] Kang Yong, [b,c] Qiao Luo, [a] Zhenlong Du, [a] Li Ma, [a] Yixin Huang, [d] Tao Zhou, [a,e] Xueping Yao, [a] Liuhong Shen, [a] Shumin Yu, [a] Junliang Deng, [a] Zhihua Ren, [a] Yong Zhang, [c] Zuoting Yan, [e] Zhicai Zuo, [a] Suizhong Cao [a]

[a]Department of Clinical Veterinary Medicine, College of Veterinary Medicine, Sichuan Agricultural University, Chengdu, China

[b]Department of Animal Husbandry & Veterinary Medicine, College of Animal Science and Technology, Chongqing Three Gorges Vocational College, Chongqing, China

[c]Department of Clinical Veterinary Medicine, College of Veterinary Medicine, Gansu Agricultural University, Lanzhou, China

[d]Institute of Biodiversity, Animal Health & Comparative Medicine, College of Medical, Veterinary & Life Sciences, University of Glasgow, Glasgow, UK

[e]Lanzhou Institute of Animal Husbandry and Veterinary Pharmaceutical, Chinese Academy of Agricultural Sciences, Lanzhou, China

These authors contributed equally: Zhengzhong Luo, Kang Yong and Qiao Luo.

Corresponding authors: Zhicai Zuo, zzcjl@126.com and Suizhong Cao, suizhongcao@sicau.edu.cn

This PDF file includes:

Figures S1 to S4

Tables S1 to S8 (Tables S5 and S7 are additional Excel files)

Supplementary Figures

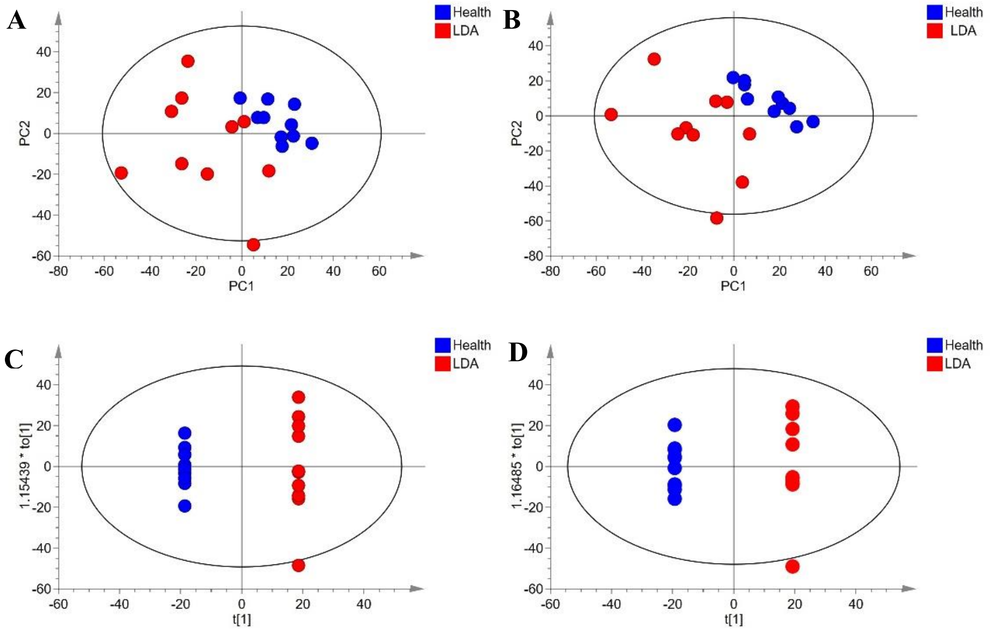

Fig.S1 Multivariate analysis for LDA and healthy groups. (A) Principal component analysis score plot for the LDA and healthy group analyzed in the positive ion mode. (B) Principal component analysis score plot for the LDA and healthy groups analyzed in the negative ion mode. (C) Orthogonal partial least square discriminant analysis of scores for the LDA and healthy groups analyzed in the positive ion mode. The following model parameter: $R^2Y = 1$, $Q^2 = 0.718$. (D) Orthogonal partial least square discriminant analysis of scores for the LDA and healthy groups analyzed in the negative ion mode. The following model parameter: $R^2Y = 1$, $Q^2 = 0.755$.

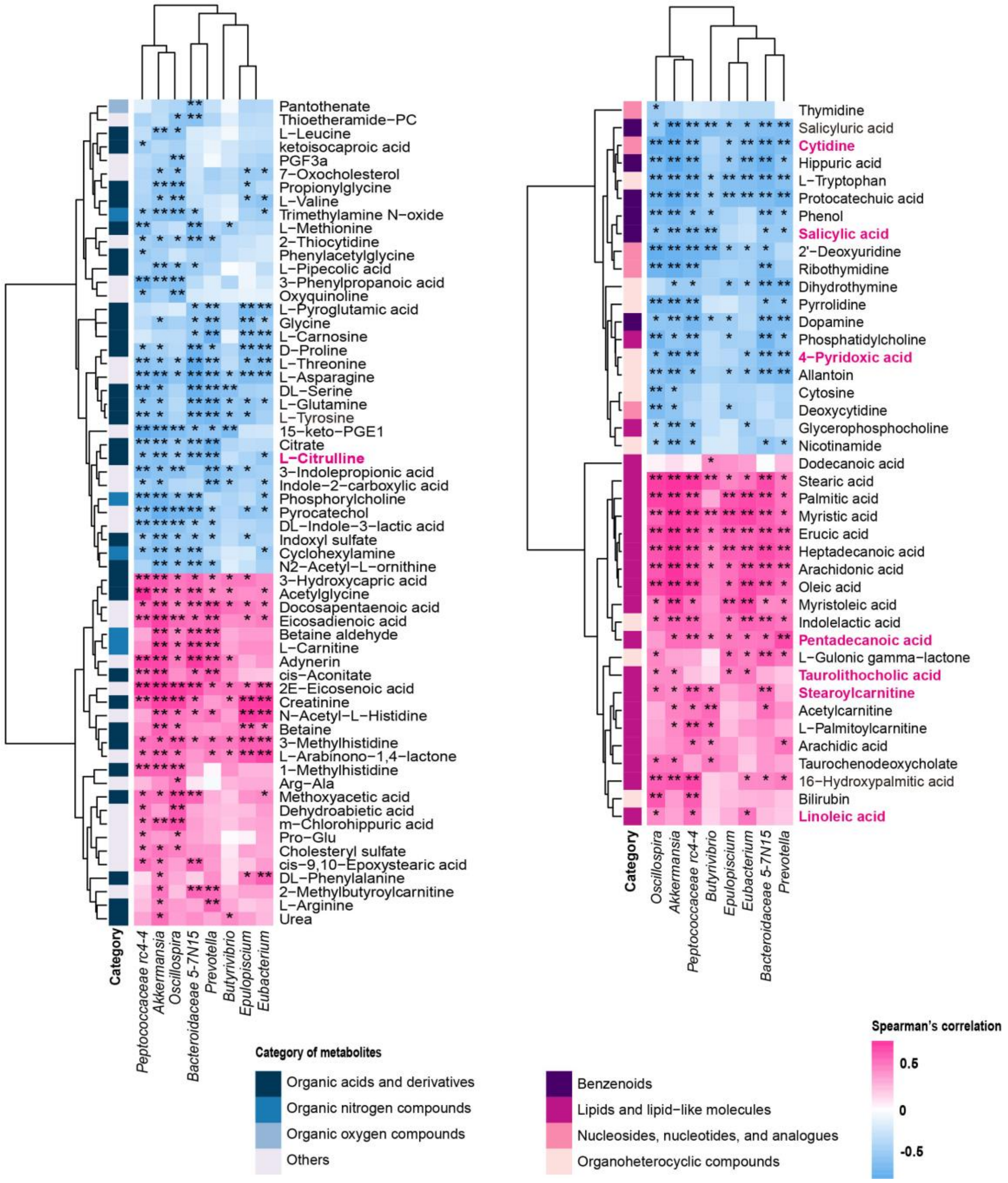

Fig.S2 Associations of LDA-related genus taxa and plasma metabolites. Heatmap depicting the relationships between the gut microbiota and plasma metabolites (n=102) that differed between healthy and LDA cows based on Spearman's rank correlation analysis. $^{*}p < 0.05$, $^{**}p < 0.01$.

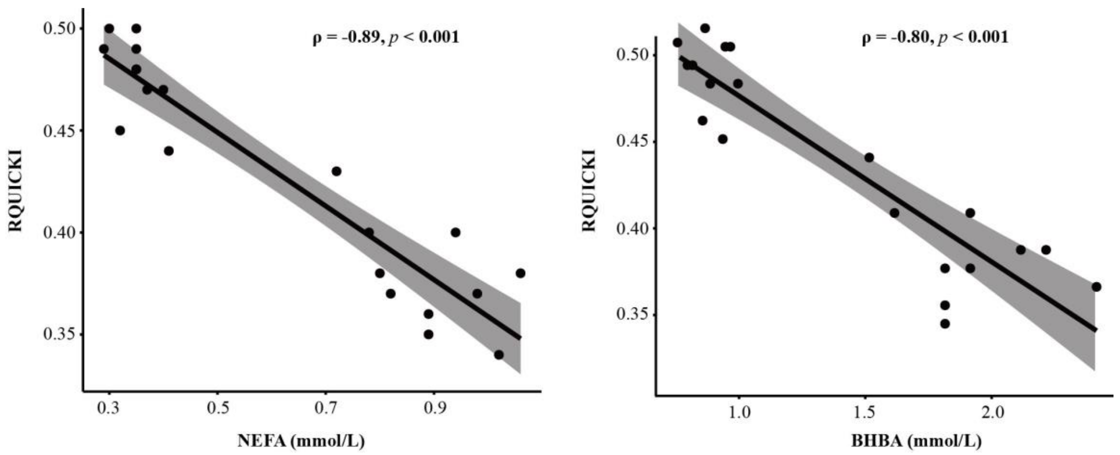

Fig.S3 The correlation plot of key metabolic markers, including revised quantitative insulin sensitivity check index (RQUICKI), non-esterified fatty acids (NEFA), and β-hydroxybutyric acid (BHBA).

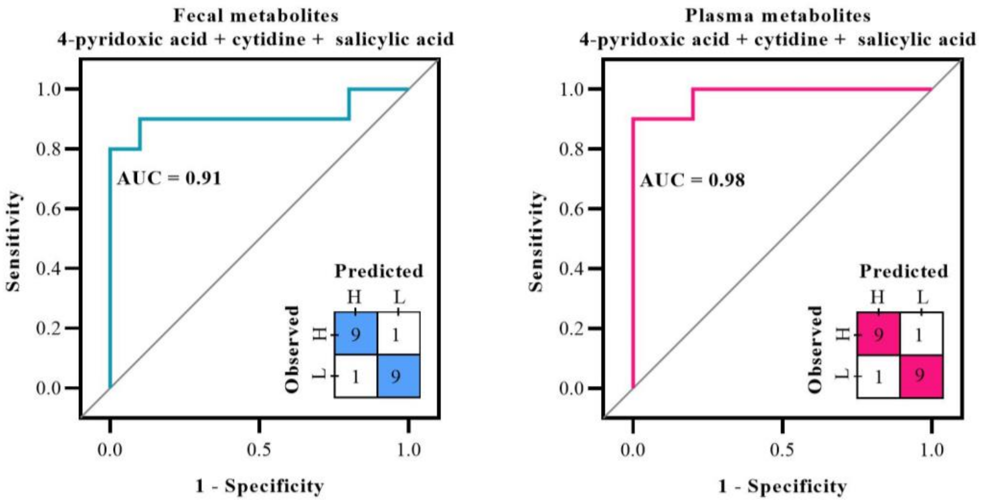

Fig.S4 Receiver operating characteristic (ROC) curve of the key metabolites in the feces and plasma. AUC, area under the curve; H, healthy cows; L, cows with LDA.

Supplemental Tables

Table S1. Ingredients and chemical composition of the diets for lactation dairy cows

Items	Levels
Ingredient, % of DM	
Alfalfa hay	22.63
Corn silage	16.52
Steam-flaked corn	20.14
Brewer's grain	5.51
Soybean meal	4.86
Extruded-soybean	7.54
Wheat bran	4.88
Cottonseed	10.19
Fat powder	0.99
Premix[a]	0.60
Calcium hydrophosphate	1.49
Calcium powder	0.59
$NaCO_3$	0.79
NaCl	0.74
Molasses	2.53
Total	100
Chemical composition, % of DM unless noted	
DM	50.36
CP	18.14
NDF	30.3
ADF	20.68
Ca	1.03
P	0.69
NE_L, Mcal/kg of DM	1.71

[a] Formulated to contain (per kilogram of premix) 1100 KIU vitamin A, 360 KIU of vitamin D_3, 6500 IU of vitamin E, 2525 mg Cu, 4200 mg Mn, 10025 mg Zn, 4150 mg Fe, 60 mg Co, 200 mg of biotin, and ≤ 10% water. NE_L, net energy for lactation.

Table S2. Information of amino acids standard in MS/MS analysis

Item	Product ID	Molecular weight	CAS number	PubChem number
Alanine	A7377	89.09	338-69-2	24891121
Arginine	A5006	174.20	74-79-3	24278014
Asparagine	A0884	132.12	70-47-3	24890464
Aspartate	A9256	133.10	56-84-8	24277948
Cystine	30200	240.30	56-89-3	329753347
Glutamate	G1251	147.13	56-86-0	24895052
Glutamine	G3126	146.14	56-85-9	24277983
Glycine	G7126	75.07	56-40-6	24895290
Histidine	H8000	155.15	71-00-1	24895816
Isoleucine	I2752	131.17	73-32-5	24896006
Leucine	L8000	131.17	61-90-5	24896488
Lysine	L5501	146.19	56-87-1	24896404
Methionine	M9625	149.21	63-68-3	24897343
Phenylalanine	78019	165.19	63-91-2	57652717
Proline	P0380	115.13	147-85-3	24898097
Serine	S4500	105.09	56-45-1	24899605
Threonine	T8625	119.12	72-19-5	24900544
Tryptophan	T0254	204.23	73-22-3	24278135
Tyrosine	T3754	181.19	60-18-4	24900164
Valine	V0500	117.15	72-18-4	24900695

Table S3. The community abundance that differed between healthy and LDA groups in phylum level

Phylum	Abundance mean in Healthy cows	Abundance mean in LDA cows	*P*-value	Fold change (LDA/Healthy cows）
Firmicutes	74.11%	73.24%	0.790	0.988
Bacteroidetes	21.30%	21.99%	0.843	1.032
Tenericutes	2.18%	1.49%	0.065	0.683
Spirochaetes	0.93%	0.39%	0.054	0.421
Proteobacteria	0.54%	1.37%	0.188	2.533
Actinobacteria	0.35%	0.04%	0.086	0.114
Saccharibacteria (TM7)	0.20%	0.06%	0.003	0.323
Verrucomicrobia	0.05%	0.93%	0.027	17.798
Euryarchaeota	0.04%	0.01%	0.042	0.225
Cyanobacteria	0.04%	0.23%	0.022	6.127
Fusobacteria	0.02%	0.13%	0.003	5.629
Fibrobacteres	0.02%	0.01%	0.342	0.307
Others	0.21%	0.11%	<0.001	0.517

Table S4. The community abundance that differed between healthy and LDA groups in genus level

Genera	Abundance mean in Healthy cows	Abundance mean in LDA cows	*P*-value	Fold change (LDA/Healthy cows)
Akkermansia	0.052%	0.932%	0.027	17.795
Anaeroplasma	0.023%	0.013%	0.076	0.554
Anaerostipes	0.023%	0.060%	0.032	2.586
Anaerovibrio	0.032%	0.067%	0.103	2.114
Arthrobacter	0.002%	0.011%	0.001	6.778
**Bacteroidaceae *5-7N15***	1.112%	2.466%	0.001	2.217
Bacteroidaceae *BF311*	0.008%	0.012%	0.475	1.474
Bacteroides	0.062%	0.052%	0.446	0.828
Bifidobacterium	0.342%	0.014%	0.073	0.039
Blautia	0.128%	0.227%	0.215	1.765
Bulleidia	0.211%	0.187%	0.520	0.889
Butyricicoccus	0.004%	0.019%	0.017	4.410
Butyrivibrio	0.047%	0.136%	0.031	2.923
Clostridiaceae *SMB53*	0.360%	0.465%	0.335	1.291
Clostridium	2.171%	2.008%	0.450	0.925
Coprobacillus	0.089%	0.060%	0.333	0.677
Coprococcus	0.591%	1.100%	0.067	1.860
Dehalobacterium	0.003%	0.006%	0.041	2.395
Dialister	0.001%	0.013%	0.221	10.742
Dorea	0.874%	0.778%	0.381	0.890
Epulopiscium	0.056%	0.149%	0.008	2.672
Erysipelotrichaceae *gut*	0.011%	0.010%	0.746	0.897
Erysipelotrichaceae *L7A-E11*	0.007%	0.018%	0.002	2.657
Erysipelotrichaceae *p-75-a5*	0.154%	0.137%	0.554	0.889
Erysipelotrichaceae *RFN20*	0.017%	0.005%	0.017	0.293
Escherichia	0.370%	0.120%	0.286	0.324
Eubacterium	0.048%	0.161%	0.021	3.382
Faecalibacterium	0.003%	0.015%	0.007	5.341
Fibrobacter	0.017%	0.005%	0.342	0.307
Fusobacterium	0.024%	0.133%	0.003	5.629
Lachnospira	0.003%	0.013%	0.012	4.945
Methanobrevibacter	0.037%	0.008%	0.042	0.225
Mogibacterium	0.055%	0.091%	0.032	1.647
Moryella	0.006%	0.037%	0.006	6.672
Oribacterium	0.002%	0.010%	0.003	4.751
Oscillospira	2.021%	3.315%	0.003	1.641
Paludibacter	0.168%	0.173%	0.889	1.031
Parabacteroides	0.071%	0.110%	0.053	1.562
Paraprevotella	0.010%	0.009%	0.798	0.902
Paraprevotellaceae *CF231*	2.068%	3.333%	0.296	1.612

Paraprevotellaceae *YRC22*	0.195%	0.104%	0.114	0.533
**Peptococcaceae *rc4-4***	0.146%	0.407%	0.001	2.793
Prevotella	0.257%	0.528%	0.004	2.054
Pseudobutyrivibrio	0.026%	0.099%	0.150	3.746
Roseburia	0.960%	1.174%	0.227	1.222
Ruminobacter	0.014%	0.048%	0.014	3.456
Ruminococcus	0.995%	1.246%	0.138	1.252
Selenomonas	0.008%	0.030%	0.033	3.753
Shuttleworthia	0.014%	0.040%	0.179	2.928
Streptococcus	0.039%	0.130%	0.162	3.320
Succiniclasticum	0.000%	0.026%	0.054	61.110
Succinivibrio	0.040%	0.404%	0.073	10.006
Treponema	0.933%	0.392%	0.055	0.421
Trueperella	0.000%	0.009%	0.023	34.057
Turicibacter	0.265%	0.284%	0.791	1.072
Veillonella	0.004%	0.011%	0.339	2.496
Unclassified	84.822%	78.590%	<0.001	0.927

Table S5. The fecal metabolites that differed between healthy and LDA groups (additional Excel files)

Table S6. KEGG pathway analysis of fecal metabolites

Pathway ID	Name	Origin	*P*-value	Metabolite ID	Metabolites
map00230	Purine metabolism	Co-Metabolism	0.000	C00559; C00575; C00212; C00330; C00387; C00147	Deoxyadenosine; Adenosine3,5-cyclicphosphate(cAMP); Adenosine; Deoxyguanosine; Guanosine; Adenine
map00591	Linoleic acid metabolism	Co-Metabolism	0.001	C01595; C06426	Linoleic acid; gamma-Linolenic acid
map00600	Sphingolipid metabolism	Co-Metabolism	0.006	C12144; C00319	Phytosphingosine; Sphingosine
map01040	Biosynthesis of unsaturated fatty acids	Co-Metabolism	0.017	C01595; C06426	Linoleic acid; gamma-Linolenic acid
map00380	Tryptophan metabolism	Co-Metabolism	0.031	C00322; C00954	2-Oxoadipicacid; Indoleacetic acid
map00240	Pyrimidine metabolism	Co-Metabolism	0.045	C00475; C00178	Cytidine; Thymine
map00220	Arginine biosynthesis	Co-Metabolism	0.121	C00327	L-Citrulline
map00750	Vitamin B6 metabolism	Co-Metabolism	0.121	C00847	4-Pyridoxic acid
map00340	Histidine metabolism	Co-Metabolism	0.169	C00785	Urocanic acid
map00300	Lysine biosynthesis	Co-Metabolism	0.174	C00322	2-Oxoadipic acid
map00120	Primary bile acid biosynthesis	Co-Metabolism	0.228	C00695	Cholic acid
map00310	Lysine degradation	Co-Metabolism	0.228	C00322	2-Oxoadipic acid
map00350	Tyrosine metabolism	Co-Metabolism	0.271	C00642	p-Hydroxyphenylacetic acid
map00680	Methane metabolism	Co-Metabolism	0.375	C00322	2-Oxoadipic acid
map00860	Porphyrin and chlorophyll metabolism	Co-Metabolism	0.452	C00500	Biliverdin
map00860	Porphyrin and chlorophyll metabolism	Microbiota	0.027	C12147; C03114	O-Phospho-L-threonine; Dimethylbenzimidazole
map00740	Riboflavin metabolism	Microbiota	0.042	C03114	Dimethylbenzimidazole

map01053	Biosynthesis of siderophore group nonribosomal peptides	Microbiota	0.042	C00805	Salicylic acid
map00780	Biotin metabolism	Microbiota	0.052	C02656	Pimelic acid
map00643	Styrene degradation	Microbiota	0.054	C02505	2-Phenylacetamide
map00626	Naphthalene degradation	Microbiota	0.064	C00805	Salicylic acid
map00400	Phenylalanine, tyrosine and tryptophan biosynthesis	Microbiota	0.081	C00296	Quinate
map00621	Dioxin degradation	Microbiota	0.081	C00805	Salicylic acid
map00622	Xylene degradation	Microbiota	0.092	C07215	2-Methylbenzoic acid
map00624	Polycyclic aromatic hydrocarbon degradation	Microbiota	0.092	C00805	Salicylic acid
map00360	Phenylalanine metabolism	Microbiota	0.109	C02505	2-Phenylacetamide
map00230	Purine metabolism	Microbiota	0.188	C02353	Adenosine2,3-cyclicmonophosphate
map00592	alpha-Linolenic acid metabolism	Host	0.017	C16300	Stearidonic acid
map00760	Nicotinate and nicotinamide metabolism	Host	0.017	C05842	N1-Methyl-2-pyridone-5-carboxamide

Table S7. The abundance of KOs that differed between healthy and LDA groups (additional Excel files)

Table S8. KOs reaction involved in leucine-isoleucine degradation and fatty acid beta-oxidation.

Ko id	Definition	Pathway	Reaction (KEGG)
k00140	Alonate-semialdehyde dehydrogenase	Valine, leucine and isoleucine degradation	propanoyl-CoA => acetyl-CoA
k00169	Pyruvate ferredoxin oxidoreductase alpha subunit	Pyruvate metabolism	pyruvate => acetyl-CoA
k00170	Pyruvate ferredoxin oxidoreductase beta subunit	Pyruvate metabolism	pyruvate => acetyl-CoA
k00171	Pyruvate ferredoxin oxidoreductase delta subunit	Pyruvate metabolism	pyruvate => acetyl-CoA
k00172	Pyruvate ferredoxin oxidoreductase gamma subunit	Pyruvate metabolism	pyruvate => acetyl-CoA
k01622	Fructose 1	Gluconeogenesis	oxaloacetate => fructose-6P
k01692	Enoyl-CoA hydratase	Fatty acid metabolism	beta-Oxidation
k01848	Methylmalonyl-CoA mutase (N-terminal domain)	Valine, leucine and isoleucine degradation	propanoyl-CoA => succinyl-CoA
k01849	Methylmalonyl-CoA mutase (C-terminal domain)	Valine, leucine and isoleucine degradation	propanoyl-CoA => succinyl-CoA
